# The Investigation of the Impact of Toxicity of Metals on Oxygen-Evolving Complex in *Spinacia oleracea*

**DOI:** 10.3390/antiox11091802

**Published:** 2022-09-13

**Authors:** Rafia Azmat, Ailyan Saleem, Waseem Ahmed, Abdul Qayyum, Hamed A. El-Serehy, Sajid Ali

**Affiliations:** 1 Department of Chemistry, University of Karachi, Karachi 75270, Pakistan; 2 Department of Horticulture, University of Haripur, Haripur 22620, Pakistan; 3 Department of Agronomy, University of Haripur, Haripur 22620, Pakistan; 4 Department of Zoology, College of Science, King Saud University, Riyadh 11451, Saudi Arabia; 5 Department of Biotechnology, Yeungnam University, Gyeongbu 712-749, Korea

**Keywords:** Copper, lead, chloride, oxygen-evolving complex

## Abstract

The current article reported the investigation of metal toxicity on the oxygen-evolving complex (OEC) in Spinacia oleracea related to depletion in chloride ion concentration, an essential part of the photosystem (II). The greenhouse experiment was conducted where *S. oleracea* was cultivated in three replicates with control plants (plants “a”) treated with tap water. Moreover, 30 ppm of Cu^2+^ ion solution and Pb^2+^ ion solution was used to irrigate the rest of the plants, labeled as plants “b” and “c”, respectively, on alternative days. Advanced technologies such as Atomic Absorption Spectrophotometry (AAS), Scanning Electron Microscopy (SEM), Energy Dispersive Spectroscopy (EDS), and UV-visible Spectrophotometry were used to monitor the essential nutrients in leaves to validate the function of the photosystem (I and II). Reduced Cl^−^ ions contents showed that both metals (Cu^2+^ and Pb^2+^) altered the essential elements of the oxygen-evolving complex (OEC) of photosystem (II), required to maintain the coordination structure of the Mn_4_CaO_5_ cluster. SEM analysis revealed the modified leaf structure of the *S. oleracea* under Cu^2+^ and Pb^2+^ accumulation due to which distorted cellular structure, reduced surface area, and the (shattered) stomatal opening compared to the plants “a” were observed. The EDS analysis of plants “b” and “c” showed high oxygen contents followed by reduced chloride contents over plants “a”, reflecting the infirmity of OEC to push out oxygen, which leads to generating oxidative stress. The lower pigment concentration in leaves of metal-contaminated plants “b” and “c” impacts carbon assimilation, which is linked to the reduced stomatal opening and influences the gaseous exchange rates. Additionally, increased contents of K^+^ and Ca^2+^ may be due to self-defense mechanisms under low chloride contents to speed up oxygen evolution to protect plants against oxidative stress. It was concluded that Cu^2+^ and Pb^2+^ metal toxicity influences essential Cl^−^ and K^+^ contents, which modify the photosystem II system; subsequently, a reduced growth rate was observed.

## 1. Introduction

Potassium (K), Nitrogen (N), Phosphorus (P), and Magnesium (Mg) are vital elements for structuring several enzymes and cell organelles, indispensable for the process of photosynthesis. Therefore, the plant observes growth reduction due to lacking these nutrients. The *Gossypium hirsutum* L. species, when grown in low concentrations of NO_3_^−^, PO_4_^2−^, or K^+^, exhibit a lower net rate of CO_2_ uptake in leaves. The relative water vapour conductance at all nutrient levels signifies the light influence on stomatal function. Though chlorophyll contents of leaves under nutrient stress were reduced, the ratio of the mesophyll surface area to the leaf area did not modify considerably.

Consequently, the less CO_2_ reduction in leaves is due to the observed low nutrient levels per unit mesophyll cell wall area. It provides new resources for assessing nutrients in CO_2_ assembling in leaves [1]. The function of photosystems I and II is vital for sustaining life; it involves carbon dioxide removal and releases O_2_ as a by-product to continue good living/breathing conditions for animals/humans. Chlorophyll, a green and environment-friendly pigment, plays a vital role in establishing the life of plants and animals. It involves eradicating the CO_2_ from the atmosphere and maintaining its level in the atmosphere. The solar radiation absorbed by photosystem II (PSII) is used to oxidize water to release O_2_ and strongly depends upon the chloride concentration [2].

The light-harvesting phenomena in photosynthesis (photosystem I) based on pigments function is referred to as chloroplast center [3,4]. While PS II (photosynthetic cyanobacteria) consists of 19 subunits of polypeptide 35 chlorophylls, two molecules of pheophytins, 11 molecules of beta carotenes, 2 molecules of plastoquinones, 3 irons in which two are of heme, and one is non-heme iron. Some ions consist of four Manganese ions, three–four Calcium ions, three Chloride ions, and one carbonate ion with 2800 water molecules encircled. The reaction center in leaves comprises two particular chlorophylls, i.e., ChlD_1_ and ChlD_2_, connected with two associated chlorophylls that are coplanar to each other, P_D1_ and P_D2_. They are the main chlorophylls that make the oxygen-evolving complex (OEC) strong enough to oxidize H_2_O into an Oxygen molecule [3]. The structure of OEC is like the cubane of the Mn_3_CaO_4_ cluster, with each metal ion in this cluster having three-µ-oxo bridges connected to another Mn ion by a mono- µ-oxo bridge in the extended region [5].

Potassium (K^2+^) is a central nutrient element needed in high concentrations for photosynthetic metabolism. The K^+^ deficiency in soil could inhibit photosynthesis and result in a yield reduction of soybean (*Glycine max* (L.) Meir). The K^+^-deficiency decreases the net photosynthetic rate (*P*_n_), transpiration rate (*T*_r_), and stomatal conductance [6]. Olesen and Andreasson [7] investigate the connection of Cl^−^ and numerous other monovalent anions in photosynthetic oxygen evolution using photosystem II membranes, drained of Cl^−^ by dialysis. In the presence of glycerol, depletion of Cl^−^ decreased 55% of the oxygen evolution rate compared to Cl^−^ present, decreasing the quantum efficiency of the reaction [8,9]. It indicated that to retain the coordination structure of the Mn_4_CaO_5_ cluster and the proton channel 2, Cl^−^ ions are required, thereby keeping the oxygen-evolving complex fully active. Wincencjusz et al. [9] proposed the hypothesis that can rationalize the requirement for Cl^−^ only on the S2-->S3 and S3-->S0 transitions that Cl^−^ required for electron transfer between Mn ions within the oxygen-evolving complex. It has been established that calcium ion acts as a “gatekeeper” that controls the water affluence of access to the OEC metalcore and lowers the activation energy barrier from the S3 to S0 transition [10,11,12,13,14,15]. Wang et al. [12] reported photoinactivation of OEC in foundation species of the angiosperm, *Phyllospadix iwatensis*, under environmental changes and human activities. They observed that the OEC of *P. iwatensis* is susceptible to photoinactivation under the light-dependent trait [13,14].

The article reports the lessening of chloride ions under metal toxicity (Cu^2+^ and Pb^2+^), which impacts oxygen evolution during photosynthesis. The *S. oleracea* (spinach) was selected for this study as (i) easy to germinate, (ii) due to their border surface area of the leaves, and (iii) relatively rich in pigments to investigate the photosystem I and II. The influence of Cu^2+^ and Pb^2+^ metal on essential nutrient ions distribution on the surface of leaves was elucidated using advanced technologies such as Scanning Electron Microscopy (SEM) in conjunction with Energy Dispersive Spectroscopy (EDS) for the first time. The function of essential nutrient ions like Cl^−^, K^+^, Ca^2+^, and Na^+^ is discussed in relation to the oxygen-evolving complex and toxicity of metals on stomatal opening.

## 2. Material and Method

### 2.1. Experimental Design

The greenhouse experiment was conducted in three sets of experimental plants in three replicates to monitor the impact of heavy metals (Cu^2+^ and Pb^2+^) on the surface morphology of the *S. oleracea* plant, followed by nutrient ions distribution. These experimental plants were marked as plants “a”, plants “b”, and plants “c” for control, Cu, and Pb contaminated plants, respectively. Plants “a” were irrigated with tap water while plants “b”, and “c” were irrigated with 250 mL of 30 ppm by the solution of Cu^2+^ (CuSO_4_) and Pb^2+^ (PbCl_2_), respectively, on alternative days. The sample of leaves from (each replicate) test plant was harvested after 6 weeks at maturity level. Each harvested tested plant was subjected to analyzed water contents, leaf area, height, and plant biomass [11]. The experiment was conducted three times to testify to the reproducibility of the same results. The chemicals used in these experiments were all of the analytical grades.

The relative water contents of leaves were examined through the method described by Barrs and Weatherley [11]. The fresh weights (FW) of the leaf sample were taken, then the samples were placed inside an oven for 1 h at 100 °C to remove complete water contents from the leaves. The dry weight (DW) of the cooled and dried sample was recorded through an analytical balance, and Relative Water Content (RWC) was determined by the following formula.

$$RWC\left(\%\right)=\left\{\frac{\left(FW-DW\right)}{\left(FW\right)}\right\}\times 100$$

The length of mature plants was recorded using a standard centimeter scale. The area of the leaves of all test plants was measured on standard centimeter graph paper, outlined using a pencil. The area of the leaf sample was then integrated.

$$area\;of\;leaf=\int_{0}^{n}area\;of\;perfect\;square+\overset{n}{\underset{0}{\int }}more\;then\;half\;filled+\int_{0}^{n}area\;of\;half\;squares$$

where *n* represents the total number of boxes on graph paper.

### 2.2. SEM and EDS Analysis of Leaf Sample

The SEM and EDS of the leaf (all sets of plants) were conducted using one leaf from each set of plants. After wearing polyethylene gloves, the leaf samples were cut using a sharp device into a disc of 10 mm. Each disc of leaf sample was then placed over a double-sided stick tape on an aluminum stub. Inside the specimen stage pan, each stub is placed on a round perforated Teflon plate, fitting it into a polycarbonate Petri dish. After creating a vacuum, the topological morphology was analyzed by a secondary electron detector. The images of SEM magnified up to (×1500) with the energy of the electron beam accelerated with 20 KV, and the probe current set at 1.00000 nA.

X-ray detector used to sense the elemental composition, characterized in the range between 0 to 10 KeV for a lifetime of 30Sec with the 8373 counts of X-rays per Sec. With the detection limit of less than 1 watt. %, elements including C, O, Na^+^, Mg^2+^, Cl^−^, K^+^, Ca^2+^, Fe, Cu^2+^ and Pb^2+^ were analyzed. The percent composition of elements was computed to renovate raw data into outcomes using the ZAF Standard less Quantitative method. That is as exact and accurate as the possible provided model assumes an initial linear relation between generated X-ray intensity and concentration of a given element. Any deviation from the initial linear relation is “corrected” by a series of multiplicative factors that interpret for the effects of atomic number (Z2014 stopping power, back-scattering factor, and X-ray production power), absorption (A), and fluorescence (F), each of which is calculated [12].

### 2.3. UV-Visible Spectroscopy

The leaves of all sets of plants were crushed separately with 80% acetone in a piston mortar to analyze several pigments, followed by centrifugation at 3000 rpm (1 h) until the complete separation of residue while the extract was transferred into a 25 mL volumetric flask and made up to the mark with the of 80% Acetone [15]. The extract was scanned on Shimadzu spectrophotometer (UV-1800A) from 200–800 nm wavelength with 80% acetone as a blank.

### 2.4. Atomic Absorption Spectroscopy

The analysis of essential elements in all three sets of plants like “a”, “b”, and “c” was conducted as reported by Citak and Tuzen [16]. Fresh leaves were harvested, weighed, washed, and dried in an oven for 2 h, followed by cooling in desiccators. After cooling, samples were grounded into a fine powder, weighted, and placed in the furnace at a temperature of 450–500 °C to change into ash. The sample was kept in a muffle furnace until the leaf samples turned into whitish ash. Then, 1 g of ash sample was taken after cooling in a desiccator and dissolved in 3.0 mL concentrated hydrochloric acid in a 100 mL beaker tagged (priorly) according to the sample. The sample was completely digested by taking 3.0 mL of deionized water in a beaker and heating it until evaporated on the hot plate. Then, it was cooled at room temperature and filtered through Whatman#1 filter paper; the filtrate was then collected in a 25.0 mL tagged volumetric flask, followed by making up with deionized water. The sample flasks were then brought to Atomic Absorption Spectrophotometer, and the signals were noted.

## 3. Results and Discussion

### 3.1. SEM and EDS Analysis of Leaves under Cu^2+^ and Pb^2+^ Toxicity

**Figure 1:**
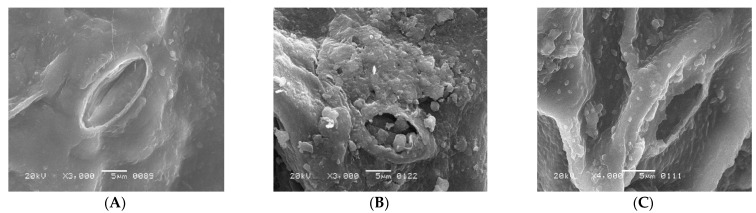
(**A**) regular shape of stomatal opening of *S. oleracea* Plant “a”, (**B**) distorted and closed shape of stomatal opening of *S. oleracea* Plant “b”, and (**C**) distorted and closed shape of stomatal opening of *S. oleracea* Plant “c”.

**Figure 2:**
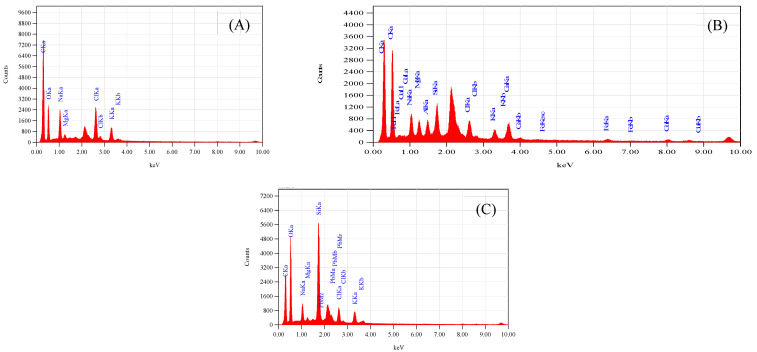
(**A**) Represents EDS spectra of S. oleracea plant “a”, (**B**) represents of plant “b”, and (**C**) represents of plant “c”.

The fixation of Cu^2+^ and Pb^2+^ on the dispersal of vital ions on the surface of leaves in *S. oleracea* was monitored using advanced technologies such as SEM, EDS, and AAS. The accumulation of Cu^2+^ and Pb^2+^ was validated through EDS of leaves, reflecting their transportation from roots to shoots and leaves. The SEM images of leaves harvested from the control plant (“a”), Cu^2+^ (plant “b”), and Pb^2+^ (plant “c”), tested plants (Figure 1A–C) exhibited a rough surface with a small stomatal opening over control. Interfering of Cu and Pb metals in the distribution of significant ions on the surface of leaves compared to control plants monitored through EDS scans (Figure 2A–C). Less content of essential nutrients such as K, Cl, Na, Fe, and C was observed, while Mg remained unaffected, and Ca was detected on the surface of leaves of metal-contaminated plants. Variation in elemental accumulation in leaves of plants “b” and “c” compared to plants “a” through EDS and AAS is linked with the surface scanning and overall analysis, respectively.

Furthermore, SEM images showed the size of stomata in leaves of the plants “a”, “b”, and “c.” The distorted surface of leaves and reduced size of the stomata indicate lower light-harvesting function followed by declined photosynthesis in plants “b” and “c”. Consequently, lower fresh and dry weights of plants “b” and “c.” were observed over plants “a” (Table 1). The distorted surface morphology of plants “b” and “c” coupled with the abridged opening of the stomata (Figure 1B,C) may recognize the unfocused photochemical and photobiological reactions. It also indicated the reduced gaseous exchange and overall photosynthetic activity. Results reported in Table 2 provide evidence of alteration of essential nutrient distribution.

### 3.2. The Function of K and Cl Ions under Stress

Potassium (K) is an essential macronutrient required in large quantities for the proper growth and reproduction of plants. It involves several biochemical processes, such as biosynthesis of the proteins, starch, water, and nutrient transportation from root to leaves. The reduced stomatal opening in plants “b” and “c” over control plant “a” (Figure 1A–C) is related to the chemical destabilizing between Cl^−^ and K^+^ because both elements are part of the processes of photosynthesis through regulating the opening and closing of stomata. In this way, plants manage the temperature by assimilating CO_2_ and transpiration of water molecules. Therefore, reduced photosynthetic activity was observed in both metal-mediated plants due to less K^+^ content on the surface of leaves [12,13,14].

The accumulation of Cu^2+^ and Pb^2+^ affects the distribution of essential micro and macronutrients on the surface of leaves that influence the electron transport chain of the plants “b” and “c.” The EDS spectra of plants “b” and “c” imitate the fewer Cl^−^ ion content, an essential part of photosystem II and numerous bioactive compounds of plants. The reduced Cl^−^ and K^+^ ion on the surface of leaves influenced the opening of guard cells during the opening and closing of stomata (Figure 1B,C). As Cl^−^ is a moveable element, its function is connected to electrical charge balance in plants as a component of photosystem II in the oxygen-evolving complex. The low intensity of chloride ions supports the generation of reactive oxygen species (ROS) due to less power of OEC [15,16,17] to push out oxygen from small stomatal openings. The current results are according to Demmig and Winter [18], who reported that in the chloroplasts center, Cl^−^ concentration is measured very efficiently under several concentrations of NaCl and understood that the O_2_ evolution is suppressed when Cl^−^ is less, which results in the trapping of triplet oxygen due to which oxidative stress is generated. The reduced stomatal opening in plants “b” and “c” also plays a vital role in generating oxidative stress, evident from high oxygen contents over plants “a.” (Figure 2A–C). The augmented oxidative stress in plants “b” and “c” can also link the less K^+^ and Cl^−^ contents with the reduced opening of the stomata on the surface of leaves, due to which singlet oxygen molecules are trapped [17]. However, high K^+^ was observed through AAS in whole leaves, which may be for the defense mechanism of the plants observed under metal accumulation. K^+^ is a mobile cation found in the guard cell responsible for stabilizing ionic balance, activating enzymes, and helping in the exchange of gasses through regulating stomata. It also improves photosynthesis, transports sugar through the phloem to different plant parts, starch, and protein synthesis, and improves crop quality [18,19]. Therefore, high K^+^ contents under metal accumulation may regulate low photosynthetic activity under low Cl^−^ ion contents, thereby increasing rate of the photosystem I and II.

### 3.3. The Toxicity of Cu^2+^ and Pb^2+^ on C Contents

The electron transport chain of plants is affected under a high concentration of Cu^2+^, while Pb^2+^ is non-essential at any concentration. However, both are toxic at high and low concentrations, respectively. The effect of both metals was observed on Carbon (C) assimilation or photosynthesis, where less C contents through SEM indicates that the lower exchange rate of both gases (O and C) in leaves of Plants “b” and “c” (Figure 2B,C) over plants “a.” The exchange rate of both gasses is directly related to the opening of stomata (Figure 1B,C), which was found to be reduced through SEM, responsible for oxidative stress and low percentage of carbon (C) assembling in leaves. The Mg^2+^ ion is an essential element of the chlorophyll molecule (Table 2), reported higher in Cu^2+^-mediated plants while less in Pb^2+^ plants (Table 3). The high concentration of Mg^2+^ in plants “b” may be due to continuous light-induced electron transport reaction from water to NADP+ but also for defense in the dark of the integrity of the water-photo oxidizing system (Photosystem II), while less in plants “c “may be linked to the false signal generated by Pb^2+^ having similar oxidation states. The EDS of the surface of the leaves of plants “b” and “c” showed the presence of Cu^2+^ and Pb^2+^ metal ions, reflecting their transportation from root to leaves compared to plants “a” (Table 3). The effect of accumulation was observed in the deactivation of phosphorylation processes followed to assist the transportation of sugars from the aerial to the underground part of a plant [19] (Table 3).

Cu^2+^ is vital for several plant enzymatic activities that run photosynthetic activity. It actively synthesizes and stabilizes chloroplast pigments and establishes plastocyanin, the chloroplast protein, tangled in the electron transport chain [20,21,22] and is an active part of the photosynthetic reaction. It is recognized that a lack of Cu^2+^ in plants appeared as abridged growth, alteration in the small younger leaves, and probable sphacelus of the apical meristem. In contrast, it is also lethal because it generates free radicals, mainly through the Fenton reaction, damaging proteins and DNA with several bioactive compounds [20,21] (Table 4). Cu^2+^ has an unusually high affinity for di-oxygen molecules (O_2_), which demonstrates why Cu^2+^ acts as a catalytic metal in numerous oxidases [23,24]. It was established that photosystem II was deactivated during the mobilization of Cu^2+^ from root to shoot. Therefore, smaller leaves turned yellow, and ultimately, the death of the plants in young states was observed.

### 3.4. Mg^2+^ a Part of Photosystem I

The Mg^2+^ ions detected by atomic absorption spectroscopy revealed that the total Mg^2+^ ion distribution (control plant = 4.00 ppm) on the surface of leaves was approximately the same as 3.98 ppm and 4.28 ppm in plants “b” and “c”, respectively (Table 4), which was according to the earlier report of Tüzen [25]. Almost the same concentration of Mg^2+^ ions in contaminated plants (Table 3 and Table 4) reflects its displacement from the porphyrin ring, supported by reduced photosynthetic activity. Therefore, the observed reduced photosynthetic activity is linked with the distorted surface of leaves and reduced absorption of solar radiation followed by low conversion of light into chemical energy. The reduced photosynthetic activity due to distorted surface and low Mg^2+^ contents impacts many primary and secondary biochemical pathways related to synthesizing vital bioactive compounds, which are valuable for plant growth [24]. Results exhibited the reduced growth of the plant due to the occurrence of these metals in leaves, which proves their interference with Mg^2+^ placement into protoporphyrinogen in chlorophyll a and b may be linked with oxidation states of both metals similar to the Mg^2+^. Similarly, it may recommend that both metals completely modify the electron transport system of photosystems I and II of chloroplast center in plants “b” and “c”, subsequently related to the reduced growth [21,25].

### 3.5. Calcium a Significant Constituent of Photosystem II

In plants, Calcium (Ca^2+^) plays a significant role in the growth of the plants and producing cell tissues. It is a non-mobile macronutrient with various functions and physiological roles in plant structure and signaling. Results of the EDS displayed that plants “b” and “c” showed the presence of Ca^2+^ while it was not observed in the control plant “a” linked to its non-mobile properties. In contrast, plants “b” and “c” showed the presence of Ca^2+^ that may be related to the solubilization process operative due to the accumulation of Cu^2+^ and Pb^2+^ on the surface of leaves. The presence of the Ca^2+^ in plants “b” and “c” may also be linked to defensive strategies adopted by these plants such as (i) it involves cell wall formation, (ii) functions in the oxygen-evolving complex to control the generation of oxidative stress under pollution, (iii) neutralizes the organic acid, (iv) acts as a detoxifying agent, (v) also associated with anions such as SO_4_ and PO_4_, and (vi) essential for seed production [26]. EDS results prove that Ca^2+^ is solubilized to protect the plants under contamination and speed up the oxygen evolution for the survival of plants as both metals interrupt the regular dispersal and role of vital micro and macronutrients [27,28].

Plants absorb nutrients from the soil, such as Mg^2+^, Ca^2+^, and Cu^2+^. The EDS and AAS revealed that Pb^2+^ also accumulated in plant “c” over plant “a” (Table 3 and Table 4). The accumulation of Pb and mobilization may be linked with the divalent state of the metal and the false signal generation by it, transported throughout the plant parts via specific protein transportation as reported in the earlier work of Maathuis and Diatloff [21]. Results showed that the divalent nature of both atoms might be involved in the accumulation, transportation, and displacement of Mg^2+^ ions from chloroplast pigments, which disturbed the overall photosynthetic activity of the plants [29] and interfered with the functions and mobilization of other minerals’ ions, reduced biomass (Table 1), distorted leaf structure, closure of the stomatal opening (Figure 1C), and reaction with other enzymes [30,31,32].

### 3.6. Fe as an Essential Factor

Iron is an essential nutrient in several metabolic processes for plant growth, chlorophyll synthesis, and maintenance of the chloroplast structure. Iron was not detected in the EDS of control plants while detected in both plants “b” and “c” (Table 3). It indicated that high Fe contents in plants “b” and “c” support the growth of the plant and regulation of the metabolic pathway, synthesis of chlorophyll coupled with respiratory cytochromes that involved in electron transfer, and the goblins that bind oxygen. The accessibility of the micronutrients on the surface of a leaf is unwaveringly linked to the solubility of water of valuable compounds. Moreover, oxidative stress in toxic plants” b” and “c” generated a Fenton reaction [23]. Fe^2+^ is readily oxidized to Fe^+3^, which is disposed to precipitate as a hydroxide even at low pH levels and immobilizes the element on the leaf surface. Though, chelating rallies Fe solubility in water, and this property can explain the lesser degree of superficial retention and, consequently, the higher uptake with chelates [23]. Piotrowska-Niczyporuk et al. [33] reported that plants usually strive by oxidative damage, catalyzed by reactive oxygen species (ROS), e.g., superoxide radical (O^2•–^), hydrogen peroxide (H_2_O_2_), and hydroxyl radical (OH•) under several environmental conditions, such as drought, chilling, high light intensity, heat, and nutrient limitations [32]. The current results reflect a high percentage of oxygen in EDS scanning that supports the generation of oxidative stress through the Fenton reaction linked to the depletion of chloride contents.

In contrast, high contents of K^+^ through atomic absorption spectroscopy are related to lowering ROS production by reducing the activity of NADPH oxidases and maintaining photosynthetic electron transport [33]. Results related to the carbon © assembling in leaves showed less content than the control plants support our hypothesis that reduced stomatal opening in plants “b” and “c” directly involved in the reduced exchange of gases due to which high contents of O_2_ and low contents^©^ (C) were observed [22]. The lower contents of C also related to low glucose formation in the primary step of the photosynthetic activity of plants related to less carbon assembling into glucose molecules; thus, the reduced weight (Table 1) of plants was observed, which was similar to the earlier reports of [20,34]. The variation observed in both advanced technologies EDS and AAS may be linked with surface analysis and total distribution in overall leaf contents (Table 4).

### 3.7. Spectral Analysis of Pigments

**Figure 3:**
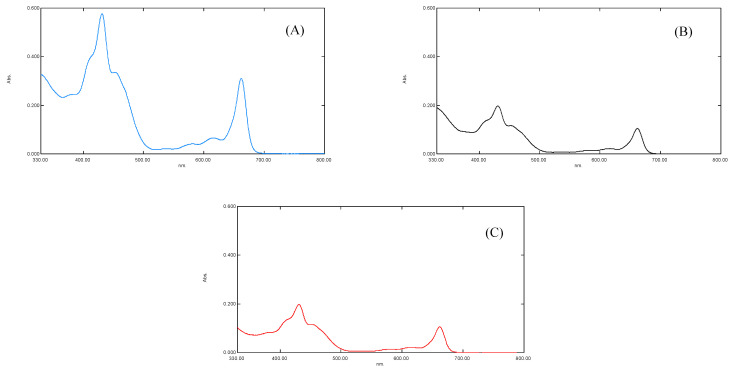
(**A**) showing UV-Vis spectra of *S. oleracea* leaves for control plant “a”, (**B**) for Copper stress plant “b”, and (**C**) for Lead stress plant “c”, respectively.

The results of the UV-Visible spectral analysis of a crude extract of plants “b” and “c” showed a reduced concentration of plant pigments, i.e., chlorophyll a, chlorophyll b, and carotenoids (antenna pigments) (Figure 3). It was noted that the maximum absorption was measured at λ_max_ of 663, 645 nm for chlorophyll a and b, 510, 480 nm for carotenoid, and 430, 617, nm for xanthophyll pigments [13]. The spectral analysis revealed that the reduced concentration of antenna pigments of plant “b” and plant “c” might be linked to the (i) increased oxidative stress (Figure 3) and (ii) the increased concentration of chemically active and non-active metals interrupted the synthesis of plant pigments. The oxidative stress may break down the photosynthetic pigments via oxidation [35].

**Figure 4:**
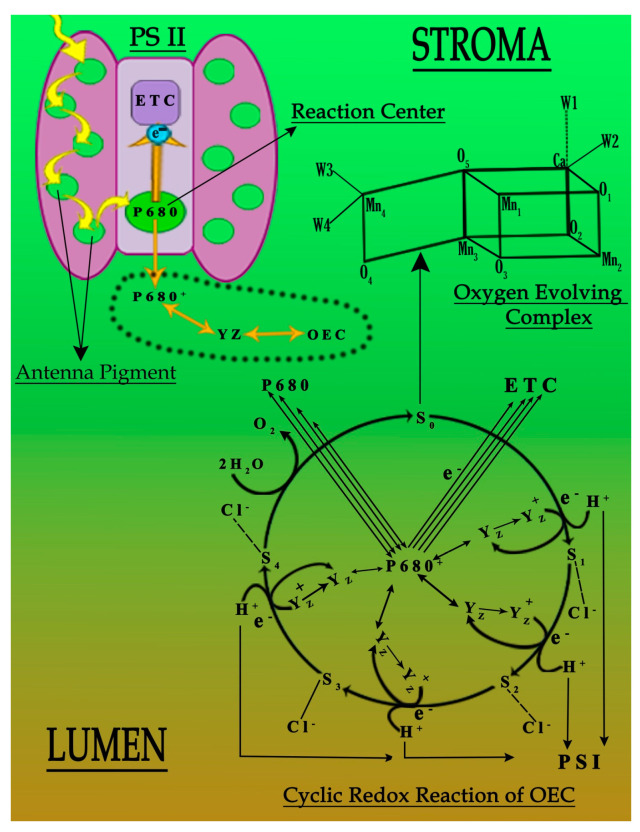
Probable deactivation of photosystem II under the influence of Cu and Pb metals.

It has been established that the function and working capability of OEC were reduced in four different ways (Figure 4). Primarily, the energy transferred to the OEC to initiate the cyclic redox reaction to oxidize water molecule into diatomic oxygen molecule decreases due to the decline in the concentration of antenna pigments (Figure 3). Secondly, the reduced chloride ion concentration affected the transition of OEC from S2 to S3. Due to its unavailability, the charge balancing of OEC was disturbed throughout its cyclic redox reaction (Table 2 and Table 3) [7,15]. Thirdly, the increased oxidative stress inside the leaf affects the production of an oxygen molecule from OEC due to the increased concentration of ROS that dominates the backward direction of the oxygen production reaction (Table 2 and Table 3). Lastly, the translocation of calcium ions from thylakoid towards the epidermis of the leaf of plant “b” and plant “c” (Table 3). This translocation reduces the strength of OEC [31,32,33,34,35,36,37], which may link to the solubilization of calcium, due to which the water-binding ability of OEC deteriorates (Figure 4). It was established that the metal interference blocked the basic movement of ions and synthesis of pigments, followed by the decrease in activities of photosystems I and II (Figure 4) [4,37].

## 4. Conclusions

It was concluded that the coordination structure of the evolving oxygen complex (Mn_4_CaO_4_) cluster was not sustained under Cu^2+^ and Pb^2+^ metal accumulation. It directly affects the proper functioning of OEC linked to reduced chloride contents followed by the generation of oxidative stress. The depletion of Cl^−^ ions concentration under Cu^2+^ and Pb^2+^ stress showed that these metals interfere at the cellular level and modify the overall essential nutrient system of the plants, due to which the oxygen-evolving complex is not sufficiently active in pulling out the oxygen from the leaves. It increases the oxygen contents, which are directly linked with oxidative stress. The lower K^+^ contents on the surface of leaves under metal contamination with reduced stomatal opening are also accountable for the generation of oxidative stress in toxic plants.

## Figures and Tables

**Table 1:** Growth regulatory system of *S. oleracea* plant “a” plants “b” and plant “c.”.

		Plant “a”	Plant “b”	Percentage (%)	Plant “c”	Percentage (%)
**Leaf area (cm^2^)**		9.98	6.146	−38.41	5.56	−44.29
**Shoot length (cm)**		7.25	7.0	−3.44	6.23	−14.07
**Root length (cm)**		3.22	2.6	−19.25	2.475	−23.14
**Water content (%)**		86 ± 0.6 **	88 ± 0.3 *	2.32	88 ± 0.1 **	2.32
**Shoot weight**	**Fresh (gm)**	1.3 ± 0.3 **	1.1 ± 0.1 **	−15.38	1.0 ± 0.1 **	−23.08
	**Dry (gm)**	0.2 ± 0.0 **	0.1 ± 0.0 **	−50	0.09 ± 0.1 **	−55
**Root weight**	**Fresh (gm)**	0.7 ± 0.0 **	0.6 ± 0.1 **	−14.28	0.5 ± 0.0 **	−28.57
	**Dry (gm)**	0.3 ± 0.1 **	0.2 ± 0.0 **	−33.33	0.2 ± 0.0 **	−33.33

±Standard deviation of three replicates, Asterisks (*) represent significant differences (*p* < 0.05); double asterisks (**) represent highly significant differences (*p* < 0.01).

**Table 2:** EDS elemental analysis of *S.* *oleracea* for control leaf plant “a”, Copper stress leaf plant “b”, and Lead stress leaf plant “c”.

Control (Plant a) Fitting Coefficient: 0.4259					
Elements	KeV	Mass %	Error %	At %	K
C	0.277	54.35	0.27	64.78	39.7680
O	0.525	31.34	0.89	28.04	29.8296
Na	1.041	6.27	0.34	3.91	10.5330
Mg	1.253	0.67	0.30	0.39	0.8965
Cl	2.621	4.83	0.22	1.95	12.6200
K	3.312	2.54	0.31	0.93	6.3528
**Copper (Plant b) Fitting coefficient: 0.4942**					
C	0.277	39.56	0.24	50.43	23.8690
O	0.525	42.33	0.58	40.51	45.5329
Na	1.041	3.86	0.34	2.57	5.0874
Mg	1.253	1.90	0.26	1.20	2.1307
Cl	2.621	1.96	0.19	0.85	4.2950
K	3.312	1.24	0.26	0.48	2.7193
Ca	3.690	2.40	0.30	0.92	5.4607
Fe	6.398	0.72	0.70	0.20	1.3525
Cu	8.040	1.57	1.46	0.38	2.7806
**Lead (Plant c) Fitting coefficient: 0.3504**					
C	0.277	34.20	0.23	44.68	14.4242
O	0.525	45.13	0.36	44.26	51.3918
Na	1.041	3.57	0.21	2.44	4.7146
Mg	1.253	0.53	0.17	0.34	0.5950
Cl	2.621	2.20	0.14	0.97	4.3874
K	3.312	1.98	0.19	0.80	4.0211
Pb	2.342	0.85	0.59	0.06	1.4086

**Table 3:** Elemental composition of leaf surface of *S.* *oleracea* plant “a”, plants “b” and plant “c” through EDS.

	Elemental % Age									
	C	O	Na	Mg	Cl	K	Ca	Fe	Cu	Pb
**Plant “a”**	54.35	31.34	6.27	0.67	4.83	2.54	-	-	-	-
**Plant “b”**	39.56	42.33	3.86	1.90	1.96	1.24	2.40	0.72	1.57	-
**Plant “c”**	34.20	45.13	3.57	0.53	2.20	1.98	-	-	-	0.85

**Table 4:** Elemental analysis of *S.* *oleracea* for control leaf plant “a”, Copper stress leaf plant “b”, and Lead stress leaf plant “c” through Atomic Absorption techniques.

Elements	Plant “a”	Plant “b”	Plant “c”
**Sodium (Na)**	24.48	24.35	24.42
**Potassium (K)**	34.94	35.51	43.25
**Magnesium (Mg)**	4	3.98	4.28
**Copper (Cu)**	0.03	2.40	0.02
**Iron (Fe)**	13.48	10.18	12.93
**Lead (Pb)**	0	0	1.67

## Data Availability

Its part of Ph.D program.
